# Early Bird Special: Quantifying Tiger Shark Predation on Albatross Fledglings at Kure Atoll

**DOI:** 10.1002/ece3.74289

**Published:** 2026-09-09

**Authors:** Caitlin Dudzik, Andrew Chin, Eva C. McClure

**Affiliations:** ^1^ College of Science and Engineering James Cook University Townsville Queensland Australia

**Keywords:** encounter rates, phenology, predator–prey interactions, resource pulse, shark–seabird interactions

## Abstract

Predator–prey interactions can be structured by predictable seasonal pulses in prey availability, yet direct evidence of temporal dynamics of predator presence, prey availability, and predation success remains scarce in marine systems. At albatross ( *Phoebastria immutabilis* and *P. nigripes* ) fledging colonies, tiger shark ( *Galeocerdo cuvier* ) aggregations are a recurring seasonal event, but the timing and dynamics of these interactions have yet to be measured. We directly quantified these interactions throughout an entire fledging season at Kure Atoll, Northwestern Hawaiian Islands. We examined temporal variation in surface‐visible predator sightings and prey abundance, environmental drivers, predation success, and seasonal patterns in predation risk. Tiger shark sightings peaked 28 days prior to peak fledgling abundance, suggesting a temporal mismatch between predator and prey, whereby sharks preferentially target early stages of prey availability when fledglings are most vulnerable, rather than periods of highest prey abundance. Environmental variables had weak and uncertain effects on both predator sightings and prey abundance, with temporal structure found as the dominant driver of observed patterns. Low strike efficiency by tiger sharks on fledglings was observed with high overall success once interactions occurred, indicating that repeated attack attempts contribute substantially to successful predation outcomes. Predation risk peaked early in the fledging season, primarily driven by temporal overlap between predator and vulnerable prey rather than changes in predator behavior or overall prey abundance. More broadly, our findings suggest that predator responses to predictable prey pulses may be shaped by variations in prey vulnerability, highlighting a key mechanism linking predator behavior to ecological patterns of predation.

## Graphical abstract

Predators are often assumed to concentrate where prey are most abundant, but the most abundant prey may not be the easiest to catch. We studied tiger sharks hunting young albatross seabirds during an albatross fledging season at Kure Atoll, Hawaii. Surprisingly, sharks arrived and largely departed before fledgling numbers peaked and predation risk was highest when inexperienced young birds first entered the water, suggesting that predators may respond more strongly to prey vulnerability than prey abundance.

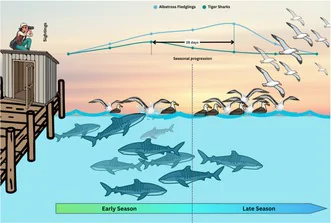

## 1 Introduction

Marine predator–prey interactions can be structured by predictable seasonal pulses in prey availability (Davoren et al. 2024), yet the temporal dynamics of predator presence, prey availability, and predation success remain poorly resolved in many marine systems (Lovell et al. 2024). Accordingly, understanding of these interactions is inferred from indirect data or broadscale modeling approaches, which may not capture fine‐scale temporal structure in ecological processes (Huse and Fiksen 2010) or fully answer temporal dynamics across entire seasonal events.

Seabird breeding colonies provide a unique opportunity to examine these dynamics due to highly synchronized fledging events (Rice and Kenyon 1962) that generate short, predictable pulses of naïve prey entering the marine environment (Meyer et al. 2009, 2010). At Kure Atoll, Northwestern Hawaiian Islands, Laysan ( *Phoebastria immutabilis* ), and black‐footed albatross ( *P. nigripes* ) fledglings enter the water over a discrete period, potentially exposing them to predation by tiger sharks ( *Galeocerdo cuvier* ), a wide‐ranging top predator known to aggregate in areas of predictable food availability (Meyer et al. 2009, 2010; Blandino et al. 2025).

Newly fledged albatrosses experience a period of heightened vulnerability as they transition from land to marine environments. Prior to departure, fledglings strengthen their wings through repeated flapping and short exploratory flights, often landing on the lagoon before returning to shore (Rice and Kenyon 1962). Individuals that leave the colony before attaining flight proficiency may become stranded on the water, where they are unable to take off effectively, become waterlogged, or succumb to predation or starvation (Rice and Kenyon 1962). Wind conditions also influence fledging outcomes, providing lift necessary for flight but occasionally resulting in adverse consequences under strong wind conditions (VanderWerf et al. 2019). Fledgling flight capability and prevailing environmental conditions together may influence the duration of exposure to marine predators.

However, it remains unclear how predator presence, prey availability, predation success, and environmental conditions are temporally structured throughout these events. For example, variability in oceanographic conditions has been proposed to influence marine predator–prey dynamics with abiotic conditions (e.g., temperature, tide, current) complicating interpretation (Schalff et al. 2014). The predictable seasonal overlap between tiger shark occurrence and albatross fledging offers a rare opportunity to explore interactions between these three near‐threatened species. In this study we quantify the timing of tiger shark sightings and albatross fledgling abundance and predation outcomes during one fledging season at Kure Atoll and investigate local environmental drivers related to predation dynamics.

Visual, direct observation surveys of tiger shark and fledgling albatross spp. interactions were conducted across the entirety of the fledging season to: (1) relate tiger shark sightings with albatross fledgling abundance and behavior, (2) investigate environmental influences on tiger shark sightings and fledgling abundance, (3) quantify tiger shark predation success, and (4) examine seasonal patterns of predation success. We hypothesize that tiger shark sightings will peak with albatross fledgling abundance increasing predator–prey overlap, that wind conditions will affect fledgling flight opportunities and time spent on the water, and that higher tide heights will increase tiger shark sightings due to improved accessibility for these large‐bodied animals in a shallow water environment.

## 2 Materials and Methods

### 2.1 Ethics Statement

Research was approved and conducted under Papahānaumokuākea Marine National Monument Conservation and Management Permit #PMNM‐2024‐001 and James Cook University Animal Ethics Permit #26A‐0015.

### 2.2 Study Site Description

**FIGURE 1:**
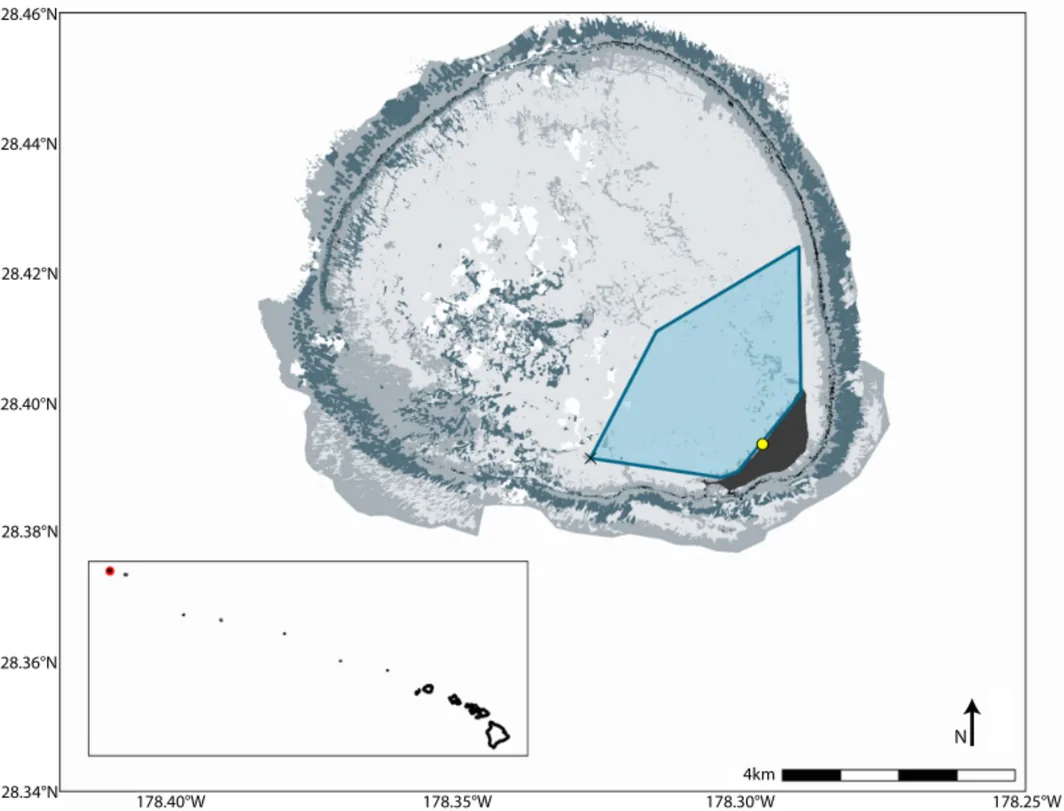
Survey site map; red point in map inset shows location of Kure Atoll within the Northwestern Hawaiian Islands; yellow point shows location of elevated observation point overlooking lagoon with shaded blue polygon displaying estimated visual survey area. Reef geomorphology derived from NOAA Pacific Islands Benthic Habitat Mapping Center (PBHMC) benthic habitat mapping products for Kure Atoll, Northwestern Hawaiian Islands (NOAA NCCOS 2003).

Kure Atoll (28°25′ N, 178°20′ W) lies at the end of the Northwestern Hawaiian Islands chain within the Papahānaumokuākea Marine National Monument, approximately 1200 km from Honolulu, Hawaii (Figure 1). The atoll is a 9.6 km wide circular lagoon, shaped by surrounding groove and spur reef that rises over 100 m from the ocean floor. Green Island (85 ha) lies within the atoll's lagoon which ranges in depth from 0 to 10 m, with most shallower than 5 m (Friedlander et al. 2009).

### 2.3 Data Collection

Predator–prey counts and interactions were investigated through daily visual surveys between 21 May 2024 and 20 September 2024 (122 days), the entire 2024 Laysan and black‐footed albatross fledging season plus 2 weeks past the last albatross fledgling sighting. Visual surveys were conducted three times a day during morning, midday, and evening hours (survey length, range: 11–157 min, mean: 55 min, median: 60 min) from an observation point (average height 6.6 m, depending on tide height) off the tip of Green Island's pier overlooking the lagoon. This provided consistent seasonal coverage across the fledging period and allowed evaluation of seasonal patterns in observed tiger shark and albatross fledgling occurrence and predator–prey dynamics. Surveys began by recording environmental data at the observation point. The lagoon was systematically scanned, aided by Celestron Nature DX binoculars (Torrance, California, USA), and all visible tiger sharks and albatross fledglings within the survey area were counted and recorded. Albatross counts included fledglings observed on the water within the survey area; individuals remaining on land or the beach were not included. New individuals entering the survey area and their movements were tracked throughout each survey to provide standardized counts while avoiding double counting. The observation area encompassed ~24% (17.2 km^2^) of the 9.6 km (Friedlander et al. 2009) wide circular lagoon (area ~72 km^2^) (Figure 1).

Environmental parameters identified as potentially affecting shark counts and albatross abundance and interactions were measured at the start of each survey (Table 1). A Kestrel 4000 Pocket Weather Tracker was used to measure air temperature, barometric pressure, wind speed, and wind direction. Wave and tide direction were recorded using visual observations against a compass. Tide height was determined using a measured reference marker against the seaward edge of the pier (depth range: 69–127 cm, mean: 96 cm, median: 97 cm). Water clarity was distinguished between clear and murky depending on whether the intersection of the break and surf zone topography was visible from the top of the observation point. Cloud cover and precipitation were evaluated using United States Air Force Manual 15–111: Surface Weather Observations (U.S. Department of the Air Force 2020).

**TABLE 1:** Environmental variables recorded at the start of each visual survey and evaluated as potential predictors of observed tiger shark sightings and albatross fledgling counts.

Variable	Unit of measure	Variable type	Justification
Air temperature	Degree Celsius	Continuous	Affects heat stress in fledglings (Mason et al. 2024)
Barometric pressure	Inches of mercury (inHg)	Continuous	Proxy for changing weather systems (Schalff et al. 2014; Udyawer et al. 2013)
Wind speed	Knots (nearest whole knot)	Continuous	Affects surface conditions; sea state, affect prey distributions, flight mechanics (Udyawer et al. 2013; Weltz et al. 2013)
Wind direction	Compass rose direction	Categorical	Flight opportunities, drives current direction (Weltz et al. 2013)
Wave direction	Compass rose direction	Categorical	Affects floating prey positioning
Tide height	Centimeters (nearest whole cm)	Continuous	Habitat accessibility (Schalff et al. 2014)
Cloud cover	Percentage	Continuous	Affects light levels, shark activity, and observer bias (Schalff et al. 2014)
Precipitation intensity	Precipitation intensity class	Categorical	Affects visibility, surface disturbance, and observer bias
Water clarity	Water clarity visual class	Categorical	Affects visibility of predator, prey, and observer

*Note:* Variables are listed with their units of measurement, data type, and ecological justification for inclusion in subsequent analyzes.

All shark sightings were recorded including identification to species. Laysan and black‐footed albatross fledgling abundance was counted at the start of each survey. To prevent double counting, the entry of new individuals into the lagoon was documented and the movement of previously counted individuals tracked throughout each survey to provide a maximum number of individuals (MaxIND) during each survey event (Sherman et al. 2018). All shark‐albatross interactions were recorded, including predation successes, predation failures, and the number of bite attempts in each interaction.

### 2.4 Data Analysis

Before statistical model fitting, we explored and processed data to address collinearity, standardized units, and variation in survey effort. Variables were examined for multicollinearity via scatterplot matrices using the *car* package (Fox and Weisberg 2019). Variables |*r*| ≥ 0.7 were considered highly collinear and not used in the analysis (Dormann et al. 2013). Where redundant variables were present, we selected continuous variables that directly measured the environmental parameter (wind speed rather than Beaufort wind scale, and Julian day rather than survey date). We standardized variables and converted data from imperial to SI units. Survey dates were converted to calendar day (Julian day) to standardize sampling across the entire study period. We converted study duration from minutes to decimal hours and log‐transformed to allow for offsetting to account for variation in observation times. All models were run using complete observations by excluding observations with any missing values.

### 2.5 Statistical Analysis

Statistical analyzes were conducted in four stages corresponding to the study aims and used complementary modeling approaches (Table 2). First, Bayesian generalized additive mixed models (GAMMs) were used to characterize seasonal temporal patterns in tiger shark sightings and albatross fledgling counts. Second, boosted regression tree (BRT) models were used to identify environmental variables associated with observed seasonal patterns. Variables identified as influential in the BRT analyzes were then used to construct Bayesian generalized linear mixed models (GLMMs) examining the effects of key environmental predictors while accounting for temporal structure. Finally, descriptive and Bayesian analyzes were used to quantify predation success and seasonal variation in predation risk.

**TABLE 2:** Overview of the analytical framework.

Aim	Ecological question	Statistical approach
1	When in the fledgling season are tiger shark sightings and fledglings most abundant?	GAMMs
2	Which environmental variables best explain predator sightings and prey abundance?	Gradient boosted regression trees followed by GLMMs
3	How often do predation attempts and successful strikes occur?	Descriptive analyzes
4	When is overall strike risk greatest?	Hierarchical ecological process model

*Note:* Four sequential ecological questions using complementary statistical models.

The specific model structures used to address each aim are summarized in Table 3. This table provides an overview of the response variables, distributions, predictor structure, and the purpose of each analysis. Detailed descriptions of each analytical approach are provided in Sections 2.5.1, 2.5.4, including model fit procedures.

**TABLE 3:** Analytical framework and model specifications used to address each study aim.

Analysis	Response	Distribution	Model structure & formula	Purpose
Seasonal phenology (Aim 1)	Tiger shark sightings	Zero‐inflated Poisson (link = “log”, link_zi = “logit”)	Julian day + survey effort offset + random intercept for survey period time of day tiger_sharks ~ s(julian_day) + offset(log(survey_length)) + (1|time_of_day)	Estimate nonlinear seasonal variation in observed tiger shark sightings while accounting for differences in survey effort and repeated daily sampling (AM, MIDDAY, PM)
	Fledgling counts	Negative binomial (link = “log”)	Julian day + survey effort offset + random intercept for survey period time of day chicks ~ s(julian_day) + offset(log(survey_length)) + (1|time_of_day)	As for tiger sharks but estimating seasonal variation in fledgling abundance
Environmental drivers (Aim 2)	Tiger shark sightings	Poisson (link = “log”)	Atmospheric pressure + wind direction + survey effort offset + random intercepts for survey period and Julian day tiger_sharks ~ pressure_inhg_c + wind_direction + offset(log(survey_hours)) + (1 | time_of_day) + (1 | julian_day)	Quantify relationships between observed shark sightings and environmental predictors identified by the BRT analyzes
	Fledgling counts	Zero‐inflated Poisson (link = “log”, link_zi = “logit”)	Tide height + wind direction + survey effort offset + random intercepts for survey period and Julian day chicks ~ tide_cm_c, + wind_direction + offset(log(survey_hours)) + (1 | time_of_day) + (1 | julian_day)	Quantify relationships between fledgling counts and environmental predictors identified by the BRT analyzes
Quantifying predation rates and interactions (Aim 3)	Predation success	—	Descriptive statistics, not formally analyzed	Summarize predator–prey interaction frequency and observed outcomes
Predator occurrence model (Aim 4)	Shark sightings, presence/absence	Bernoulli (link = “logit”)	Standardized Julian day + standardized log survey duration + random intercept for survey period time of day	Estimate nonlinear seasonal variation in the probability of observing tiger sharks
Prey occurrence model (Aim 4)	Fledgling presence/absence	Bernoulli (link = “logit”)	Standardized Julian day + standardized log survey duration + random intercept for survey period time of day	Estimate nonlinear seasonal variation in the probability of fledgling presence
Conditional strike probability (Aim 4)	Successful strike	Binomial (link = “complementary log–log”)	Standardized Julian day + standardized log survey duration + random intercept for survey period time of day	Estimate the probability of a successful strike conditional on observed predator–prey co‐occurrence
Overall strike probability (Aim 4)	Derived posterior probability	—	Derived from the product of posterior predictions from the predator occurrence, prey occurrence and conditional strike models	Estimate the overall probability of a strike occurring during a survey

*Note:* Simplified model structures are given for clarity. Smooth terms (e.g., “s(julian_day)”) represent penalized regression splines used to model nonlinear relationships with Julian day. Continuous predictors were mean‐centered prior to analysis, distinguished here by “_c.” Survey effort was included either as a log‐transformed offset (count models) or a standardized log‐transformed covariate (binary models). Random intercepts accounted for repeated observations within survey periods and, where indicated, Julian day. Common Bayesian fitting settings are summarized in Table 5.

All Bayesian models were fit using the *brms* package (Bürkner 2017) with the *CmdStan* backend (Stan Development Team 2023). Weakly informative priors were assigned based on the median and median absolute deviation of each response variable to prevent unrealistic estimates while allowing flexibility in ecological relationships (Lemoine 2019). Model performance was evaluated using prior and posterior predictive checks to assess whether model predictions were consistent with observed data distributions (Gelman et al. 2020; McElreath 2020), and convergence was assessed using trace plots, effective sample sizes, and $\hat{R}$ diagnostics (Vehtari et al. 2021). Residual diagnostics were evaluated using the DHARMa package (Hartig 2024). All models were well mixed and converged on a stable posterior ($\hat{R}$ < 1.01) with adequate effective sample sizes.

#### 2.5.1 Seasonal Phenology in Tiger Shark Observations and Albatross Fledgling Counts (Aim 1)

To characterize the seasonal phenology of tiger shark sightings and albatross fledgling abundance, we fit GAMMs to the survey data using R (R Core Team 2025). The objective of these models was to estimate the timing and shape of seasonal patterns, rather than identify environmental drivers of variation. Environmental predictors were examined separately in Aim 2. Tiger shark sightings and fledgling abundance were modeled separately against Julian day, with Julian day included with a smooth term to account for the nonlinear temporal trend in species observations (Table 3). Survey effort (in decimal hours) was included as a log‐offset to account for the influence variation in survey duration might have on tiger shark sightings and albatross abundance. Survey time of day (AM, MIDDAY, PM) was included as a random effect to account for consistent differences among the three standardized daily sampling periods and repeated sampling structure within days. The seasonal timing of median predictions was identified as the Julian day when cumulative predicted abundance reached 50% of the season total, with 95% credible intervals.

#### 2.5.2 Environmental Drivers of Seasonal Dynamics (Aim 2)

Using gradient boosted regression tree (BRT) models, we explored environmental variables driving tiger shark sightings and albatross fledgling abundance separately (De'ath 2007; Elith et al. 2008; Hastie et al. 2009). Environmental variables collected during each survey included air temperature, atmospheric pressure, wind speed and direction, tide height, wave direction, cloud cover, precipitation intensity, and water clarity (Table 1), survey time of day, and time of season (Julian day) with an offset of log‐transformed survey duration included to account for sampling effort variation. Water temperature was not measured during this study due to equipment availability, though we acknowledge having access to these data would be ideal. Time of day is known to be an important temporal driver of shark abundance at islands with albatross fledglings at French Frigate Shoals (Blandino et al. 2025). Here, we account for an effect of time of day while testing for effects of other environmental variables such as wind direction, wind speed, air temperature. All BRT model analyzes were conducted in R using the *gbm* package (Ridgeway and GBM Developers 2026). Separate Poisson‐distributed (loss function) models were developed for tiger shark count predictions and fledgling abundance using environmental covariates and survey effort as an offset. Modeled parameters, including learning rate, interaction depth, minimum observations per node, and optional number of trees determined by using threefold cross‐validation (Elith et al. 2008), were tuned independently for each model (Table 4). Final model settings resulted in 943 optimal trees for the tiger shark model and 1472 optimal trees for the fledgling model. Confidence intervals and relative influence for the partial effects of each covariate were extracted from the optimum number of trees, estimated by bootstrapping 1000 times. Variables were considered of significant relative influence if they exceeded a threshold calculated by 100/12 variables.

**FIGURE 2:**
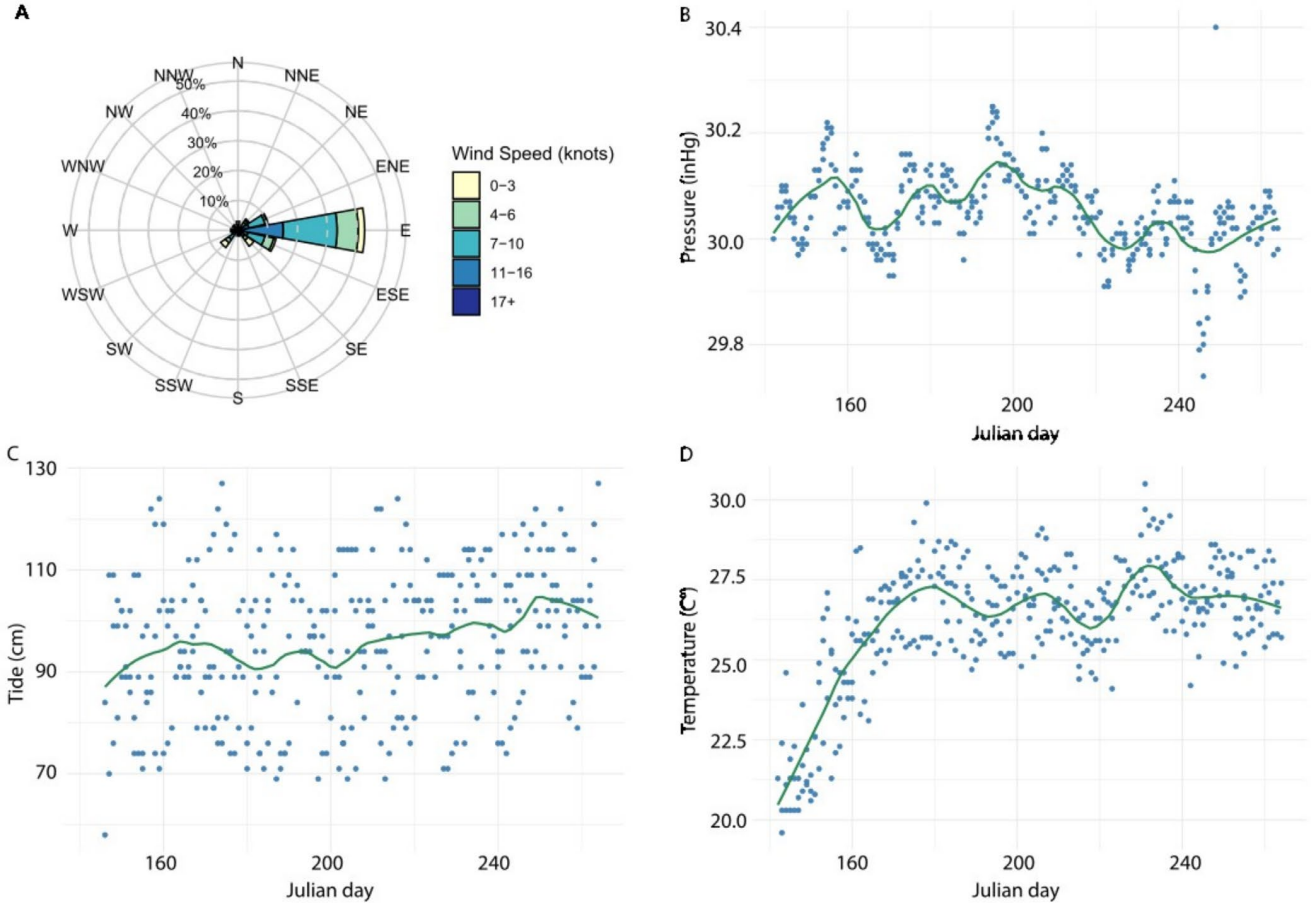
Environmental conditions recorded during tiger shark survey effort across the study season. (A) Wind rose showing the number of surveys conducted under different wind directions and wind speeds (knots). Wind direction was variable throughout the study period but was dominated by easterly winds (~42% of surveys). (B) Atmospheric pressure (inHg) by Julian day, (C) tide height (cm) by Julian day, and (D) air temperature (°C) by Julian day. In panels B–D, green lines smoothly connect observations sequentially to visually illustrate cyclical variation in environmental conditions through time.

We examined environmental predictors identified as important in the BRT analyzes for temporal patterning prior to GLMM construction (Figure 2). Although not statistically collinear, several variables displayed cyclical variation across Julian day. Top‐ranked BRT predictors were retained for GLMM analysis, while acknowledging that temporal structure within the environmental conditions may add complexity to the interpretation of independent environmental variables.

Based on the BRT analysis, the highest‐ranking environmental predictors for each response variable were subsequently evaluated using Bayesian GLMMs. We modeled tiger shark sightings against atmospheric pressure and wind direction. Fledgling abundance was modeled against wind direction and tide height. While Julian day was identified as having a high relative influence on both shark counts and fledgling abundance, it was not included as a fixed effect in GLMMs because the relationship of tiger shark counts and fledgling abundance with Julian day was explicitly evaluated in seasonal analyzes (Aim 1).

Instead, Julian day and survey time of day were included as random effects to account for hierarchical sampling structure and nonindependence among observations associated with repeated surveys (Harrison 2014; Wood 2017). Including these terms allowed the models to account for variation associated with repeated sampling across survey dates and daily survey periods, while reducing unexplained variation. Thus, both tiger shark sightings and fledgling abundance were modeled against two predictor variables only to prevent overfitting and simplify interpretation of results (Table 3). Continuous variables of tide height and atmospheric pressure were centered to improve model parameter interpretability (i.e., intercept and slope) and improve model convergence, with wind direction modeled as a categorical variable. Survey effort was included as a log‐offset to account for the variation in survey duration, assuming a proportional relationship between survey effort and observed individuals. Prediction grids were generated across the observed range of Julian day, with survey duration held constant at 60 min (the approximate median survey length) to standardize sampling effort during model prediction. The models were fit and assessed using standard procedures detailed in Section 2.5.1 and Table 5.

**TABLE 4:** Parameter settings for gradient boosted regression tree (BRT) models used to identify environmental predictors associated with observed tiger shark counts and albatross fledgling counts.

Parameter	Tiger shark model	Fledgling model
Distribution (loss function)	Poisson	Poisson
Learning rate	0.0002	0.01
Tree complexity	10,000	10,000
Interaction depth	2	3
Bag fraction	0.5	0.5
n.trees	943	1472
Train Fraction	1	1
n.minobsinnode	10	5
Cross‐validation folds	3	3
Model structure	gbm(tiger_sharks ~ julian_day + wind_direction + tide_cm + temp_c + pressure_inhg + wind_knots + cloud_cover + precipitation + wave_direction + tide_direction + water_clarity + time_of_day + offset(log(survey_hours))	gbm(chicks ~ julian_day + wind_direction + tide_cm + temp_c + pressure_inhg + wind_knots + cloud_cover + precipitation + wave_direction + tide_direction + water_clarity + time_of_day + offset(log(survey_hours))

*Note:* Separate models were fitted for tiger sharks and albatross fledglings using environmental variables, survey duration as an effort offset, and Julian day to account for seasonal variation. Distribution = response distribution used for model fitting; Learning rate = contribution of each tree to the overall model; Tree complexity = maximum number of trees evaluated during model fitting; Interaction depth = maximum interaction order between predictor variables; Bag fraction = proportion of data randomly selected to build each tree; n.trees = number of trees selected using cross‐validation; Train fraction = proportion of observations used for model fitting; n.minobsinnode = minimum observations required in terminal nodes; Cross‐validation folds = number of subsets used to estimate predictive performance; Code = model formula used for analysis.

**TABLE 5:** Common Bayesian model fitting settings.

Setting	Study default	Exceptions	Justification
Software	brms (CmdStan)	—	brms package (Bürkner 2017) with cmdstand backend (Stan Development Team 2023)
Priors	Weakly informative	—	Prevent unrealistic estimates while capturing ecological relationships (Lemoine 2019)
Chains	3	Strike models: 4	Number of independent Markov Chain Monte Carlo (MCMC) chains
Iterations	8000	Strike models: 10,000	Total MCMC iterations per chain
Warm‐up	1000	Strike models: 2000	Iterations discarded during model adaptation before posterior sampling
Thinning	5	Strike models: 7	Interval at which posterior samples were retained
adapt_delta	0.95	Strike models: 0.99	Reduce divergent transitions and improve sampling efficiency (Stan Development Team 2023)
max_treedepth	Default (10)	Strike models: 15	Allows for more complex trajectories
MCMC Diagnostics	$\hat{R}$^, ESS, trace plots, posterior predictive checks	Applied to all models	Thresholds for $\hat{R}$ < 1.01 (Vehtari et al. 2021), bulk and tail Estimated Sample Size > 100 times number of chains (Stan Development Team, n.d.), autocorrelation plots (Stan Development Team 2023), posterior predictive checks and residual diagnostics via the DHARMa package (Hartig 2024)

#### 2.5.3 Predation Rates and Interactions (Aim 3)

We estimated predation success using descriptive statistics as the number of interaction events observed (*n* = 26) was insufficient for robust statistical modeling of predictors of success. Accordingly, analyzes were limited to summary measures of interaction frequency and observed outcomes.

#### 2.5.4 Seasonal Variation in Predation Success (Aim 4)

Using a hierarchical ecological process model framework, we modeled predation probability. Overall strike probability was estimated by independently modeling prey occurrence, predator occurrence, and conditional strike probability, then combined through posterior predictions (Kéry and Royle 2020; McElreath 2020). Specifically, in the first stage, prey (albatross fledglings) presence and predator (tiger shark) sightings were modeled independently as binary (presence/absence) functions of Julian day to quantify temporal variation in encounter opportunity. In the second stage, the probability of strikes by tiger sharks on fledglings was modeled conditionally on the co‐occurrence of sharks and fledglings (i.e., both sharks and fledglings needed to be observed as present).

For the shark and fledgling presence models, we used Bayesian binomial regression models with logit links, with centered Julian day included as a smooth term to allow for nonlinear temporal trends (Table 3). Survey duration was included as a log‐transformed covariate to account for variation in sampling effort. Time of day was included as a random intercept to account for repeated sampling within survey periods. For the behavioral model, strike probability was modeled using a Bayesian binomial regression with a complementary log–log link, which allowed for modeling event probabilities arising from an underlying rate process (McCullagh and Nelder 1989). Temporal variation in strikes was modeled using a smooth function of centered Julian day; survey duration was included as a log‐transformed covariate to account for variation in sampling effort, and fledgling presence was included as a fixed effect. Time of day was included as a random intercept to account for repeated sampling within survey periods.

The strike model was fit only to observations in which both tiger sharks and fledglings were observed, ensuring that behavioral processes were estimated independently of variation in encounter opportunity (i.e., observations lacking either a predator or prey could not result in a strike and were therefore excluded from the behavioral model). Instead, these cases were accounted for in earlier stages of the modeling framework, where prey and predator presence were modeled explicitly. Consequently, the overall probability of a strike event during a survey approaches zero when either prey or predators are absent through the multiplicative combination of model components. Posterior predictions from each stage were combined to estimate the overall probability of a strike occurring during a survey:

$$P\left(\text{strike}\right)=P\left(\text{birds}\right)\times P\left(\text{sharks}\right)\times P\left(\text{strike}\;|\;\text{birds},\text{sharks}\right)$$

Predictions were generated at the population level from 1000 posterior draws of each model (excluding random effects) with survey duration held constant at its median value (60 min). Peaks in probability were extracted for the presence of fledglings and sharks and overall strike probability, but not for the conditional model for which there was no discernable peak.

#### 2.5.5 Data Visualization and Map Production

All figures were generated using the *ggplot2* package (Wickham 2016). The map of Kure Atoll was produced in R (R Core Team 2025) using packages *sf*, *dplyr*, *ggspatial*, *cowplot*, and *rnaturalearth* and reef geomorphology and benthic habitat shapefiles (kure_class_geog and kure_cover_geog) (NOAA National Centers for Coastal Ocean Science 2003). Detailed habitat classifications (HABCLASS) were aggregated into broader functional habitat classes relevant to predator–prey interactions, including reef edge, reef, hardbottom, rubble, sand, deepwater, and land habitats. The “surf” class was treated as a proxy for reef‐edge habitat. Survey platform location and estimated survey‐area polygons, comparing observation references with aerial maps, were overlaid using simple feature (sf) objects referenced to WGS84 geographic coordinates (EPSG:4326). A manually defined graticule was added to provide geographic reference. The inset map of the Hawaiian Archipelago was generated using Natural Earth coastline data cropped to the coordinate extent of the Hawaiian Islands.

## 3 Results

### 3.1 Effort

Between 21 May 2024 and 20 September 2024, 359 surveys were conducted over 122 days with 325:57 h of direct observation.

### 3.2 Seasonal Phenology in Tiger Shark Observations and Albatross Fledgling Counts (Aim 1)

A total of 1564 albatross spp. fledglings were observed and recorded in the lagoon, representing 4.29% of the all‐island nest count conducted in December 2023 (Agreement on the Conservation of Albatrosses and Petrels 2024) and 7.29% of the all‐island chick count conducted six months later in June 2024 (N. Worcester, personal communication, June 20, 2026). Across all observations, 247 sharks were recorded, including 43 tiger sharks, 162 Galapagos sharks, and 42 unknown shark species large enough to discount smaller species such as the Galapagos shark but not definitively identifiable as tiger sharks.

**FIGURE 3:**
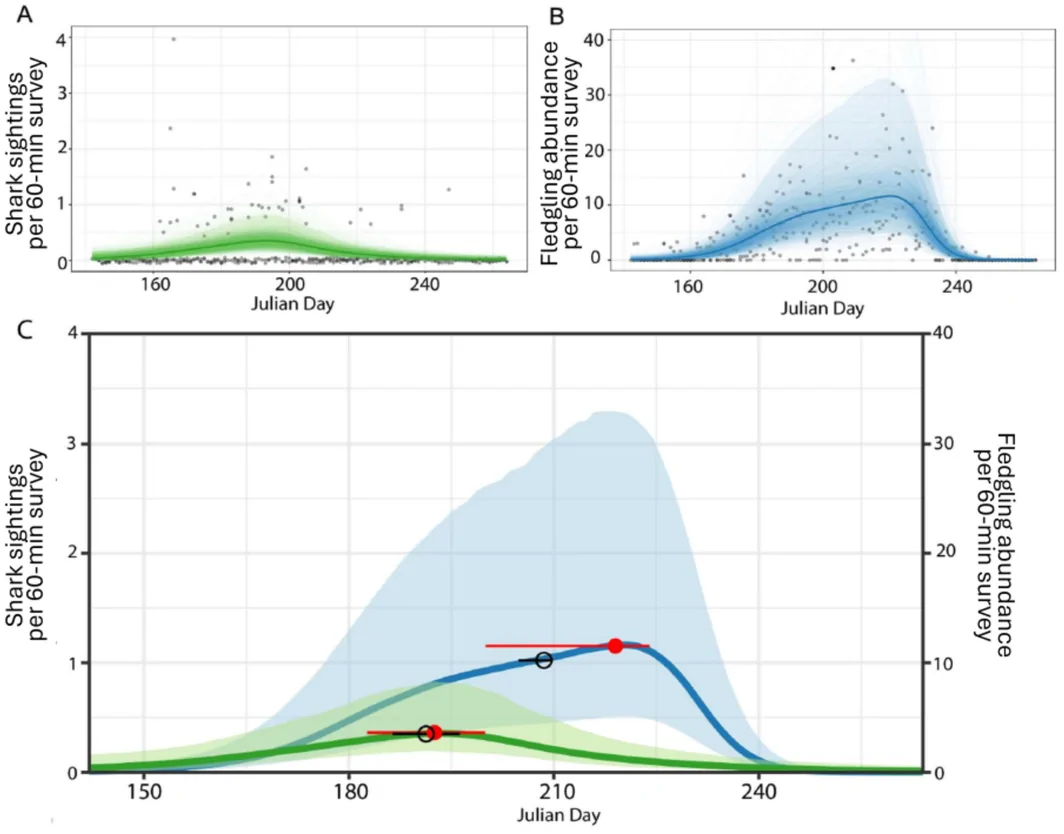
(A) Seasonal sightings of tiger sharks and (B) albatross fledglings across the fledging season. (C) Combined seasonal abundance with tiger shark sightings (green; left axis) and fledgling abundance (blue; right scaled axis) shown with model‐estimated trends with 95% credibility interval ribbons reflecting uncertainty in estimates. Points denote estimated peak counts (red) and median counts (black) with horizontal credibility intervals display uncertainty in timing estimates.

Bayesian GAMMs showed a gradual increase in albatross fledgling abundance from 143 Julian day, with fledgling numbers reaching estimated peak abundance at 220 Julian days (07 Aug) (95% credible interval (CrI): 200–224 Julian days) (Figure 3B), 77 days from the first fledgling observed in the lagoon. This demonstrates a slow rise in fledgling numbers early in the fledging season, with strong evidence for median abundance occurring at 208 Julian days (26 July) (95% CrI: 204–210 Julian days), 12 days before peak abundance. Fledgling abundance declined rapidly after peak abundance with the last fledgling observed in the lagoon 30 days after peak abundance. The smooth function of Julian day, the added fixed effect, explained a moderate proportion of variation in fledgling abundance. The marginal R2 (Rm), representing variance explained by the fixed effects alone, was 0.331 (95% Crl: 0.088–0.674), with conditional R2 (Rc), representing variance explained by both fixed and random effects, was 0.409 (95% Crl: 0.315–0.514). These results indicate that seasonal structure contributed substantially to variation in fledgling numbers.

Tiger shark sightings exhibited a symmetrical pattern with the season, with peak sightings occurring on 192 Julian days (95% CrI: 182–200 Julian days) and median sightings occurring on 191 Julian days, 10 July and 09 July, respectively (95% CrI: 186–197) (Figure 3A). This suggests a relatively consistent rate of arrival and departure of surface‐visible tiger sharks during the survey period around the seasonal peak. Seasonal structure, as denoted by the smooth Julian day fixed effect, explained small to moderate variation of tiger shark survey sightings (Rm = 0.127, 95% CrI: 0.021–0.341; Rc = 0.135, 95% CrI: 0.054–0.239).

Peak tiger shark survey sightings occurred 28 days before albatross fledgling abundance peaked, while median shark sightings occurred 17 days earlier than that of fledglings, indicating sharks were most abundant before most fledglings were available for predation (Figure 3C). Shark sightings were already declining before fledgling abundance reached its seasonal maximum.

### 3.3 Environmental Drivers of Seasonal Dynamics (Aim 2)

**FIGURE 4:**
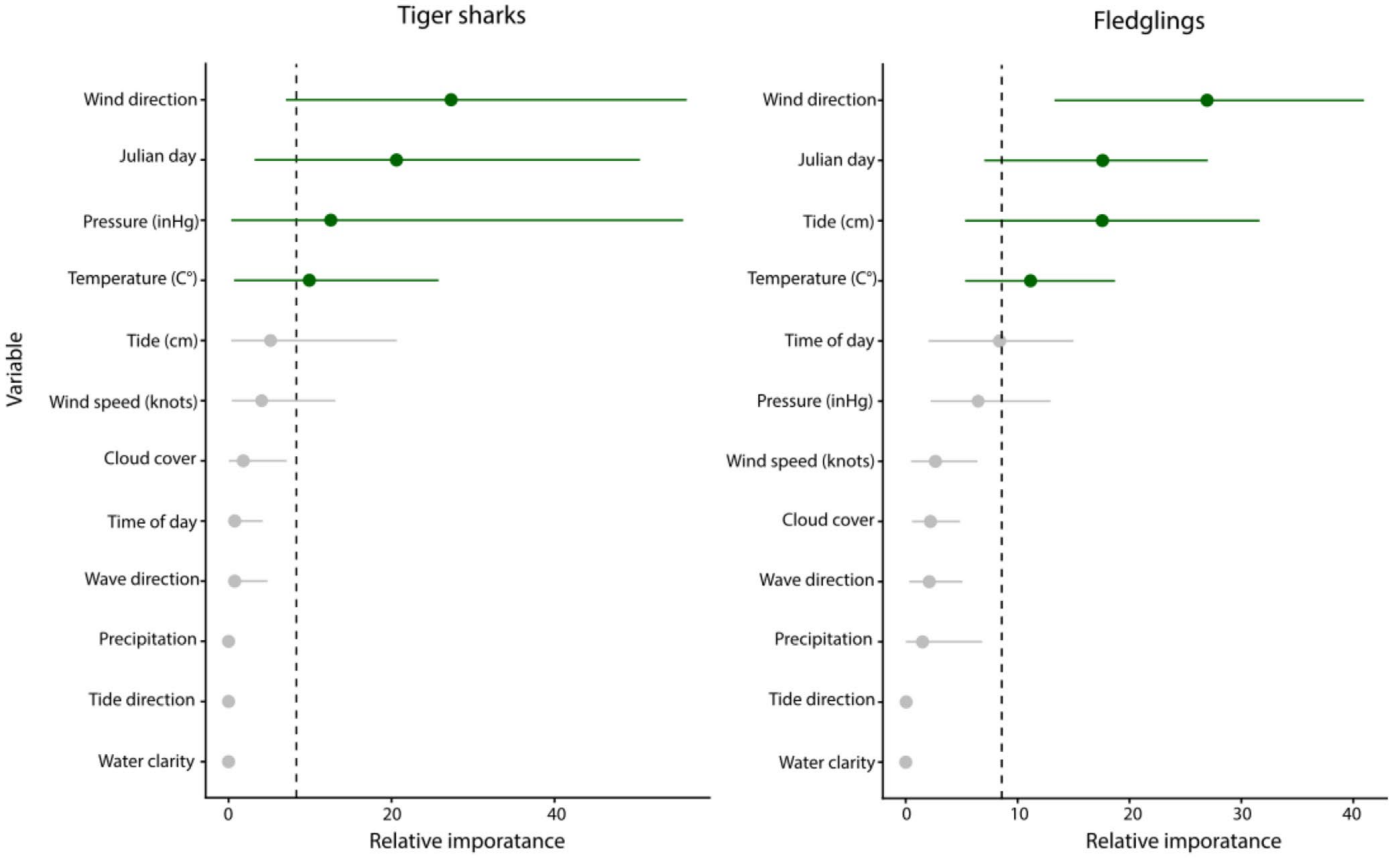
Relative influence of environmental and temporal predictors of tiger shark sightings and albatross spp. fledgling abundance based on 1000 bootstrapped boosted regression tree (BRT) models. Vertical dashed lines indicate the threshold separating influential predictors from those contributing less than expected background variation. Gray points and 95% confidence intervals represent predictors below the influence threshold, whereas green points and confidence intervals represent predictors above the threshold. (Tiger sharks) Relative influence of predictors contributing to BRT predictions of tiger shark counts, including wind direction, Julian day, atmospheric pressure, and other environmental variables. (Fledglings) Relative influence of predictors contributing to BRT predictions of albatross fledgling counts, including wind direction, Julian day, tide height, and other environmental variables.

Bootstrapped gradient boosted regression tree (BRT) models identified key environmental predictors of tiger shark sightings; wind direction was the most influential predictor in the BRT model (27.3%), followed by Julian day (20.6%), atmospheric pressure (12.5%), and air temperature (9.9%) (Figure 4). However, uncertainty around predictor effects was high, and the remaining environmental variables contributed relatively little to model predictions. For albatross fledgling abundance in the lagoon. Wind direction (23.4%), Julian day (18.0%), and tide height (17.3%) were the most influential variables (Figure 4). The remaining eight predictor variables contributed less to the model, with six of those falling below the 9.1% influence threshold.

**FIGURE 5:**
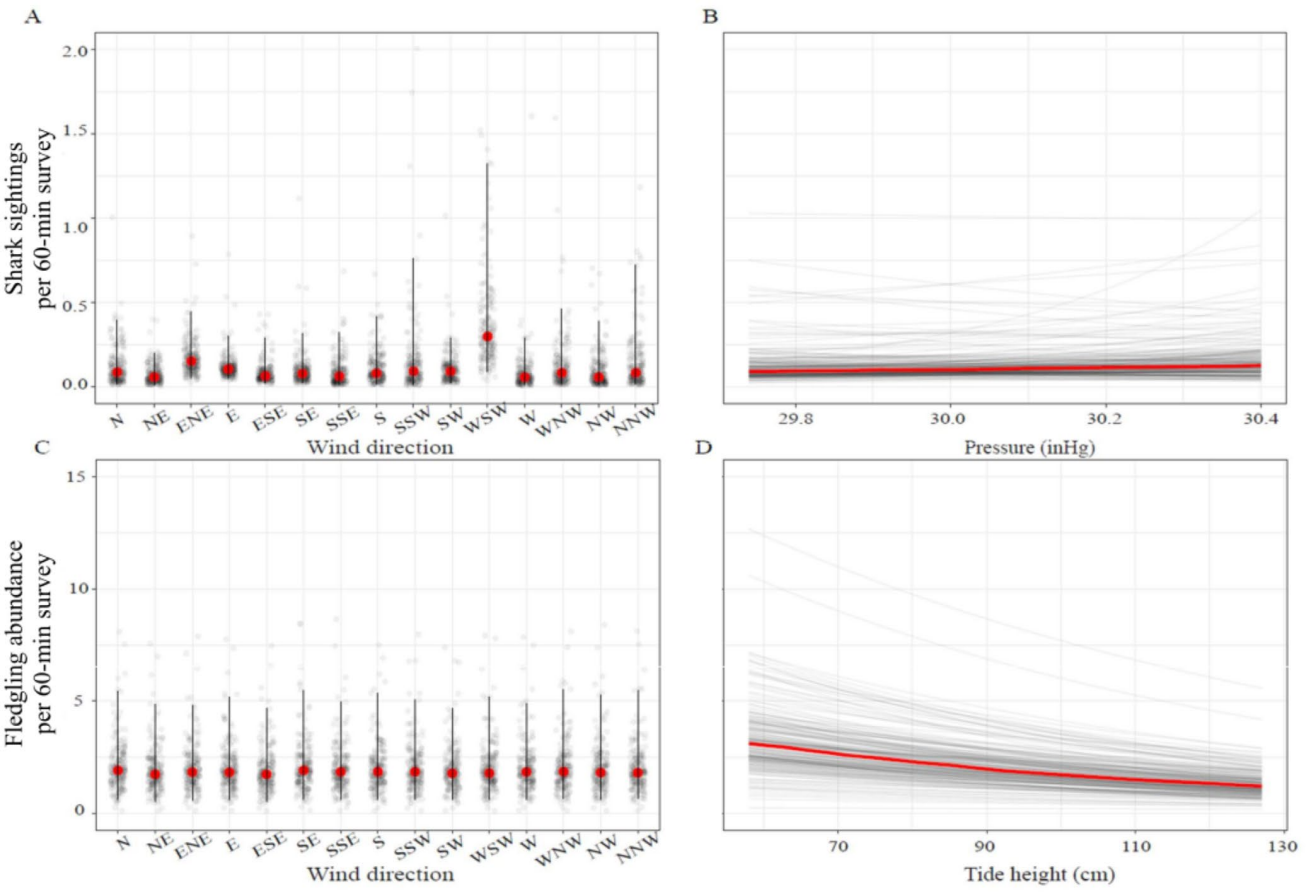
Partial effects of environmental variables on tiger shark counts (A, B) and albatross fledgling abundance (C, D) from Bayesian GLMMs. Gray points and lines represent draws from posterior distributions with red points and trend lines the estimated marginal means.

While BRTs identified several variables with relatively high predictive influence, the GLMMs indicated weak support for strong effects on albatross fledgling abundance and tiger shark sightings. This likely reflects the different roles of the modeling approaches, with BRT machine learning approaches emphasizing prediction, and GLMMs evaluating the strength of the relationship and uncertainty around estimated effects in this system. Environmental predictors identified as most influential variables identified in the BRT model showed only weak relationships with effects on tiger shark sightings in the Bayesian GLMMs. Wind direction (Figure 5A) and atmospheric pressure (Figure 5B) showed little variation in predictions' influence on tiger shark sightings across the observed predictor ranges. Bayesian marginal R^2^ indicated that fixed environmental effects explained very little in shark sighting variation (Rm = 0.029, 95% CI: 0.001–0.152), where conditional model effects were higher (Rc = 0.38, 95% CI: 0.161–0.595), indicating stronger variation attributed to the temporal structure of the random effects, Julian day and time of day. Similarly, for albatross fledglings, wind direction (Figure 5C) and tide height (Figure 5D) revealed minimal variation in estimated abundance across the variable ranges. Bayesian marginal *R* ^2^ indicated that fixed environmental effects explained almost no variation in abundance (*R*_m_ = 0.009, 95% confidence interval (CI): 0.000–0.050), where conditional model fit was considerably higher (*R*_c_: 0.775, 95% CI: 0.699–0.840), highlighting strong temporal contributions explained by Julian day and time of day random effects on the model.

### 3.4 Predation Rates and Interactions (Aim 3)

**FIGURE 6:**
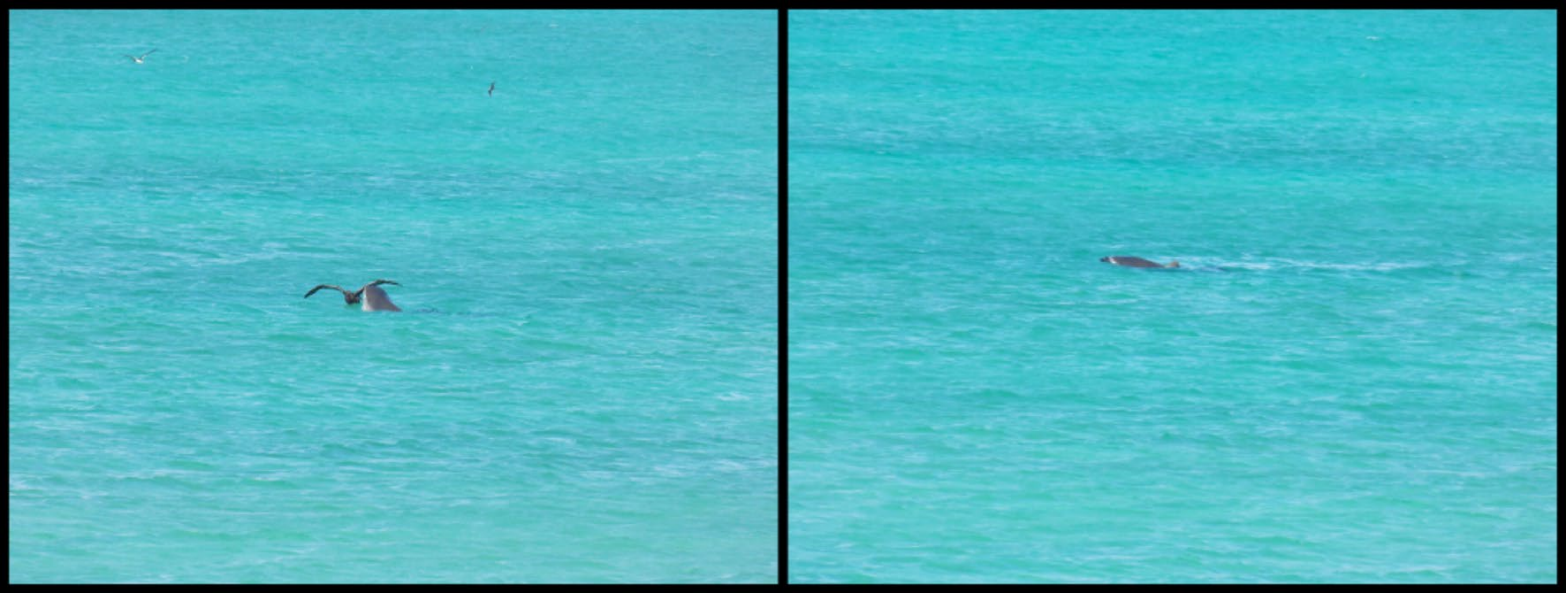
Photo series of Interaction 9, which resulted in successful predation of black‐footed albatross fledgling by tiger shark.

During observations over the fledging season, a total of 26 tiger shark‐albatross spp. interactions were documented. Twenty interactions (76.9%) resulted in ultimate successful predation events (Table 6 and Figure 6).

**TABLE 6:** Summary of the 26 observed interactions between tiger sharks and Laysan or black‐footed albatross fledglings recorded during the 2024 fledging season.

Interaction	Date	Time of day	Tiger shark count	Fledgling count	Bite attempts	Successful bites	Bite success rate	Ultimate predation success?
1	13‐Jun	MIDDAY	2	2	1	1	100%	Yes
2	14‐Jun	MIDDAY	1	2	2	1	50%	Yes
3	14‐Jun	P.M.	1	1	2	1	50%	Yes
4	15‐Jun	MIDDAY	1	3	1	1	100%	Yes
5	20‐Jun	MIDDAY	1	7	4	1	25%	Yes
6	20‐Jun	MIDDAY	1	7	3	1	33.33%	Yes
7	30‐Jun	A.M.	1	17	4	1	25%	Yes
8	1‐Jul	A.M.	1	12	2	0	0%	No
9	1‐Jul	A.M.	1	12	1	1	100%	Yes
10	6‐Jul	A.M.	1	16	3	1	33.33%	Yes
11	11‐Jul	P.M.	1	10	1	1	100%	Yes
12	12‐Jul	A.M.	1	9	2	1	50%	Yes
13	13‐Jul	A.M.	3	25	4	1	25%	No
14	13‐Jul	A.M.	3	25	4	1	25%	Yes
15	16‐Jul	P.M.	1	2	4	1	25%	Yes
16	21‐Jul	A.M.	2	65	1	1	100%	Yes
17	21‐Jul	A.M.	2	65	2	0	0%	No
18	21‐Jul	A.M.	2	65	1	0	0%	No
19	21‐Jul	A.M.	2	65	3	1	33.33%	Yes
20	22‐Jul	P.M.	1	5	1	0	0%	No
21	23‐Jul	P.M.	1	10	1	1	100%	Yes
22	5‐Aug	P.M.	1	9	4	1	25%	Yes
23	8‐Aug	P.M.	1	5	7	1	14.29%	Yes
24	11‐Aug	A.M.	1	45	4	0	0%	No
25	20‐Aug	P.M.	1	6	1	1	100%	Yes
26	20‐Aug	P.M.	1	6	1	1	100%	Yes

*Note:* For each interaction, the survey date, survey period, number of tiger sharks and fledglings present, number of bite attempts, successful bites, bite success rate, and whether the interaction resulted in successful predation are reported. Bite success rate was calculated as the proportion of successful bites relative to the total number of bite attempts during each interaction.

**FIGURE 7:**
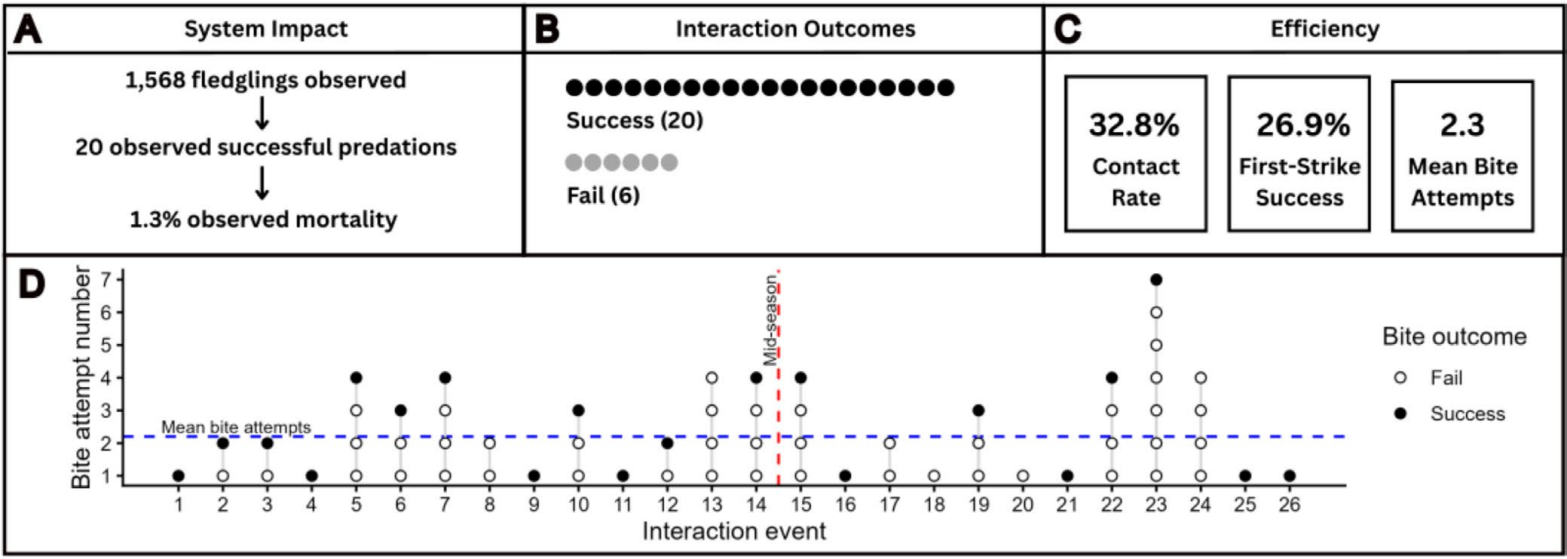
Summary of tiger shark predation on albatross fledglings. (A) System‐level impact showing total fledglings observed, predation events, and overall mortality. (B) Interaction outcomes across 26 shark–prey encounters, distinguishing successful and failed predation events. (C) Summary of predation efficiency, including contact rate, first strike success, and mean bite attempts per interaction. (D) Bite attempt sequences across 26 tiger shark–albatross interactions, showing failed (open circles) and successful (filled circles) bite attempts per interaction. The dashed red line indicates the mid‐season boundary. The dashed blue line indicates the mean number of bite attempts observed across all interactions.

This represents 1.3% mortality of the fledglings counted within the survey area throughout the season (*n* = 1568). In most interactions, tiger sharks had a low strike success rate and required repeated approaches prior to ultimate successful or failed predation, with only a 32.8% contact rate across all interactions (Figure 7).

### 3.5 Seasonal Variation in Predation Success (Aim 4)

**FIGURE 8:**
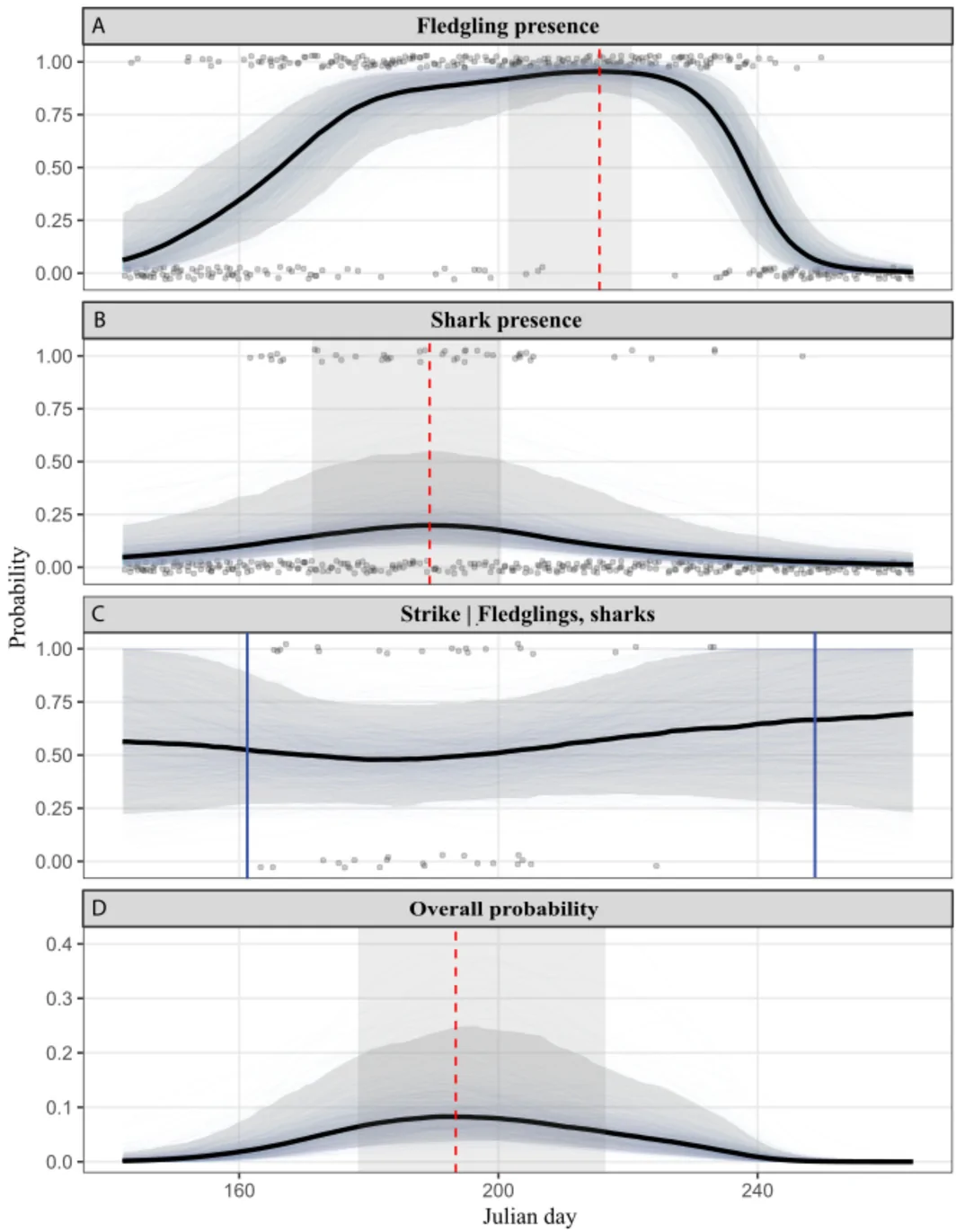
Seasonal patterns in prey presence, predator presence, and strike probability. (A) Predicted probability of observing albatross fledglings during a survey. (B) Predicted probability of observing tiger sharks during a survey. (C) Predicted probability of a tiger shark strike, conditional on both predator and prey being present. (D) Overall predicted probability of a tiger shark strike, combining predator presence, prey presence, and conditional strike probability. Points represent observed presence (1) or absence (0) for individual surveys. Lines show posterior median predictions and shaded ribbons indicate 95% credible intervals. Red dashed vertical lines indicate peak predicted probability; gray boxes show the corresponding 95% credible intervals. Solid navy vertical lines indicate the temporal bounds of confirmed tiger shark observations.

Seasonal dynamics in predation appear primarily driven by temporal overlap of prey and predator occurrence, rather than variation in strike behavior (Figure 8). Consistent with the prey abundance and predator sighting patterns with time, the strike probability model showed shark presence peaking earlier than fledgling presence, at Julian day 189 (95% CI: 171–200) and Julian day 216 (95% CI: 201–221), respectively. Accordingly, the overall probability of a strike (the multiplied effect of prey availability, predator occurrence, and interaction probability conditional on predator–prey presence) peaked at approximately Julian day 193 (95% CI: 178–217), shortly after the peak in shark presence and approximately 3 weeks before maximum fledgling availability.

In contrast, the modeled probability of a strike conditional on the presence of both fledglings and sharks gave no clear unimodal peak with time (Figure 8C). Strike probability remained broadly similar across the season, with only a weak indication of a decline early in the season followed by a slight increase after approximately Julian day 200. Uncertainty increased at the temporal boundaries of Julian day where there was no or low occurrence of sharks and low event frequency.

## 4 Discussion

This study provides unique, quantitative insights into predator–prey interactions from regular and repeated direct observations of tiger sharks preying upon fledgling albatross during a predictable prey pulse event. These observations provide direct quantified evidence of the timing of predator sightings and prey peak and median abundance and provide insights into the variables that may explain these observed patterns, as well as predation success rates and the changes in predation event probability throughout a predictable prey pulse event.

### 4.1 Seasonal Phenology in Tiger Shark Observations and Albatross Fledgling Counts (Aim 1)

Contrary to the hypothesis that shark sightings in the lagoon would coincide with fledgling abundance, we found a clear temporal offset between peak predator sightings and prey abundance. Tiger shark sightings peaked nearly a month, 28 days, before albatross fledglings' abundance peaked, with shark median observations also occurring earlier in the season. The temporal imbalance between predator and prey suggests that tiger shark arrival is not coupled with maximum prey abundance but instead overlaps with the early stages of fledgling presence in the lagoon.

This early‐season overlap may reflect a period of increased vulnerability, as early in the fledging season, fledglings entering the water are inexperienced and likely more vulnerable to predation (Rice and Kenyon 1962). The decline in observed shark sightings as fledgling numbers were still rising further supports this interpretation and is consistent with movement studies of some tiger shark individuals at prey pulse events, including albatross fledging in the Hawaiian chain (Meyer et al. 2010) and turtle nesting in the western Pacific (Fitzpatrick et al. 2012). Rather than remaining for the duration of an entire prey pulse, sharks may preferentially target periods when prey is easier to capture, rather than when prey is most abundant and more difficult to catch.

This offset highlights that the predator–prey interactions in this system are temporally structured but not synchronized. Consequently, predation risk may be disproportionately concentrated during the initial stages of fledging, when predator and prey distributions overlap with higher sightings of predators and relatively low prey abundance.

Tiger shark occurrence at Kure Atoll should be interpreted within the broader context of tiger shark movement ecology in the Northwestern Hawaiian Islands. Telemetry studies have demonstrated that tiger sharks can move extensively among islands within the region, including movements involving Kure Atoll (Papastamatiou et al. 2013). At French Frigate Shoals, tiger sharks have been documented aggregating in association with seasonal pulses of albatross fledglings, although telemetry studies also indicate that some individuals remain in these habitats year‐round (Lowe et al. 2006; Meyer et al. 2010; Papastamatiou et al. 2013; Blandino et al. 2025). The seasonal pattern observed in surface‐visible tiger sharks in this study may be indicative of arrival into and departure from the lagoon, as supported by modeling and observer knowledge of the study area.

### 4.2 Environmental Drivers of Seasonal Dynamics (Aim 2)

Environmental variables exhibited relatively weak relationships with observed tiger shark and albatross fledgling counts, with variation being largely structured by temporal factors. Although the BRT models identified several environmental variables with relatively high influence, these effects were not strongly supported by Bayesian GLMMs. In addition, environmental variables were not collinear with Julian day; several variables, including tide height and pressure, showed cyclical patterns across both the diel cycle and the fledging season. Accordingly, temporal predictors may have captured broader seasonal dynamics within the system, limiting the ability to isolate independent environmental associations.

Wind direction had the highest relative influence in the tiger shark BRT model; however, this relationship should be interpreted cautiously. BRTs identify variables associated with predictive performance but do not establish causal relationships, and the subsequent Bayesian GLMM provided little evidence for a strong effect of wind direction on observed tiger shark sightings. Consequently, we do not interpret wind direction as a direct or indirect driver of tiger shark occurrence in the study area.

Albatross fledgling abundance on the lagoon is likely driven by biological timing associated with development and fledging schedules (Rice and Kenyon 1962; Sprague and Breuner 2010), rather than short‐term environmental variability. Similarly, tiger shark sightings appear not to be linked to environmental conditions but to the predictability of a foraging area (Holland et al. 1999; Heithaus et al. 2007; Meyer et al. 2009), including the seasonal prey pulse albatross fledglings deliver (Lowe et al. 2006; Meyer et al. 2010).

The weak influence of environmental predictors examined in Aim 2 reinforces that the temporal structure identified in Aim 1 is likely the primary driver of both tiger shark sightings and albatross fledgling abundance patterns in this system.

### 4.3 Predation Rates and Interactions (Aim 3)

Tiger shark predation on albatross fledglings was characterized by low strike efficiency but high overall success. Strike efficiency of tiger shark attack attempts was relatively low (32.8%) across all observed interactions, with multiple attempts often required before successful capture. Only 19.2% of interactions (5 of 26 interactions) resulted in successful predation from the first strike, demonstrating that most successful predation events required repeated attack attempts.

Despite this low strike success, overall predation success was high once an interaction began. Of the 26 observed interactions, 76.9% (20 interactions) resulted in successful predation, suggesting high mortality of fledglings despite low encounter frequency. The distinction between low strike success and high overall predation success suggests that persistence is a key determinant of hunting success in this system.

Evidence of unsuccessful initial attacks has been documented in other systems. Heithaus (2006) reported high frequencies of tiger shark‐inflicted bite scars on dolphins, indicating failed predation attempts are common, with an estimated unsuccessful shark attack rate between 11%–13% of dolphins in Shark Bay, Western Australia. Additionally, tiger shark foraging behavior observations suggest these predators rely on stealth and opportunistic targeting rather than early presence detection, high‐speed chases, and scenarios that require high maneuverability, which lessen the chances of predation success (Heithaus et al. 2002). These findings are consistent with these results where patterns of repeated attack attempts increase the likelihood of predation success.

Tiger sharks may benefit from their temporal overlap with early‐stage fledglings who retain substantial downy plumage and have limited flight performance (Rice and Kenyon 1962), reducing their ability to leave the water rapidly following an unsuccessful attack. Under these conditions, repeated attack attempts increase the likelihood of eventual predation success.

The temporal mismatch identified in Aim 1 suggests that tiger sharks overlap with fledglings during an early developmental stage characterized by reduced flight capability, which may contribute to high predation success observed once interactions occur. At the population level, fledgling mortality from shark predation was low (1.3% of observed fledglings), suggesting tiger shark predation alone is unlikely to influence overall colony size substantially. However, as surveys were conducted three times a day, this estimate of predation is likely conservative. Nevertheless, the ecological significance of this interaction may not be reflected solely by population‐level mortality. The temporal overlap between tiger sharks and newly fledged albatrosses creates a period of increased predator–prey encounter opportunity.

Although tiger sharks may create a period of elevated predation risk for fledglings after entering the lagoon, this risk is unlikely to influence fledgling timing or habitat‐use decisions. Albatross fledglings must transition to the marine environment to develop independent foraging ability, and the timing of fledging is driven primarily by developmental readiness (Rice and Kenyon 1962). Therefore, tiger shark presence likely does not represent a behavioral cue influencing fledgling decisions to enter the water. Instead, any increased susceptibility to predation during early fledging likely reflects the limited flight capability and escape capacity of recently fledged birds once they encounter a tiger shark within the lagoon.

### 4.4 Seasonal Variation in Predation Success (Aim 4)

Temporal variation in predation risk was primarily driven by seasonal overlap between tiger sharks and albatross fledglings, rather than changes in strike behavior or temporal changes in the probability of attack once predator–prey encounters occurred. The overall probability of a strike peaked early in the fledging season, shortly after peak sightings of tiger sharks and before peak fledgling abundance, indicating that predation risk is highest when predators and vulnerable prey co‐occur (Carrier et al. 2010).

In contrast, the probability of a strike given the presence of both sharks and fledglings showed no strong temporal pattern and remained relatively consistent throughout the season. This suggests that seasonal variation in predation risk was not driven by changes in tiger shark attack probability, but instead by changes in the likelihood of predator–prey overlap (Heithaus and Dill 2006). Early in the season, higher tiger shark sightings coincided with fewer available fledglings, whereas later in the season, declining shark sightings overlapped with increasing fledgling abundance. Thus, variation in strike probability appears to be shaped by the timing of predator and prey occurrence rather than changes in predator behavior once encounters occur. Tiger sharks are generalist, opportunistic predators (Ferreira et al. 2017), and risk to their prey is determined by when their presence overlaps in time.

While the conditional strike model did show slight temporal variation, there was no clear peak or pattern in strike probability. Additionally, increased uncertainty at the temporal boundaries on Julian day where there were no observed sharks and low frequency of events coupled with the lack of a distinct predation peak may reflect the combined effect of rare strike events limiting statistical power. We therefore interpreted predictions in these regions cautiously.

### 4.5 Limitations

Direct observation methods have several limitations that we considered during interpretation of results. Estimates of tiger shark observations and albatross fledgling numbers may be underestimated due to visual constraints including sun glare, cloud reflection, and sea state on the lagoon's surface. Furthermore, lightning events precluded six surveys, creating minor gaps in temporal coverage. Although a consistent survey approach by a single observer minimized variability in effort and detection, these factors may have introduced some observational bias.

This study was limited to a single fledging season at one location and therefore does not capture interannual variability or spatial differences in predator–prey dynamics. The 26 documented predator–prey interactions represent direct observations of predation dynamics during the fledging season at Kure Atoll and provide a quantitative baseline for evaluating predator–prey interactions during future fledging events at this and other locations. Additional years of standardized monitoring would be valuable for assessing the consistency of seasonal predator–prey overlap and predation patterns across interannual variation in environmental conditions and prey availability.

Using observed shark detections could represent a source of uncertainty. Surveys were restricted to the lagoon visible from the Green Island observation point and therefore did not encompass the entirety of Kure Atoll or the full spatial distribution of tiger sharks within the atoll. Furthermore, the absence of observed tiger sharks from the survey does not necessarily indicate the absence of sharks from Kure Atoll, as individuals may have been outside the survey area, too far below the water's surface to be observed, or active outside survey periods. Surveying during three standardized daylight periods allowed us to account for variation among survey periods but did not characterize continuous diel patterns in tiger shark habitat use. Accordingly, the models using observed shark presence likely underestimate predator occurrence and should be interpreted as representing observed surface‐visible tiger shark occurrence within the survey area rather than true tiger shark abundance or atoll‐wide presence.

Finally, while this study focused primarily on the temporal drivers of sightings and predation risk, environmental conditions may still influence behavior and vulnerability at finer scales. Factors such as wind speed may aid fledglings in evasive maneuvers to escape predation (Udyawer et al. 2013; Weltz et al. 2013) and tide height (Schalff et al. 2014), water clarity, and glare may play important roles in successful predation attempts by sharks. Future work incorporating these variables with a larger dataset would help discover the relative roles behavioral, environmental, and sensory mechanisms play in shaping predation outcomes.

### 4.6 Implications/Broader Significance

These results not only contribute to our understanding of ecological patterns in predator–prey dynamics but also highlight implications for how ecological relationships inferred from indirect observations are used to predict ecological processes. In marine systems specifically, dietary studies (DeCrosta 1981; Stevens et al. 2000), stock assessments (Hutchings and Myer 1994; Myers et al. 1996), and ecosystem models (Grubbs et al. 2016) have been employed in place of direct observations to quantify predator–prey interactions. Although these approaches are valuable, their outputs can strongly shape management narratives when assumptions are untested.

Previous work demonstrates how influential ecological pattern models can become in management contexts. For example, trophic cascade models and stock models contributed to interpretations surrounding the Atlantic cod collapse influencing understanding of ecosystem restructuring and fisheries decline (Hutchings and Myer 1994; Myers et al. 1996). Similarly, the “Save the Bay, Eat a Ray” public campaign emerged from the hypothesis that declines in large sharks eased cownose rays from predation pressures, resulting in increased shellfish predation; however, this trophic cascade hypothesis was later challenged because of limited empirical support and uncertainty in the underlying ecological process assumptions (Grubbs et al. 2016).

Across ecological applications, model outputs can be sensitive to parameter uncertainty, structural assumptions, and limited mechanistic realism, which can result in misleading predictions that can have serious policy implications (Getz et al. 2018). A foundational study in marine trophic cascades suggests that the removal of tiger sharks may restructure marine food webs through indirect interactions across multiple trophic levels by increasing a mid‐level consumer (seabirds) and could have devastating effects on lower trophic levels (tuna) (Stevens et al. 2000). These model hypotheses are informed, in part, by a temporal snapshot of dietary data from stomach contents analysis of 27 tiger sharks (DeCrosta 1981), which suggested that tiger sharks are key predators of fledglings.

In this context, direct observational data collected over ecologically relevant temporal scales can aid model parameterization and improve model reliability. Direct observations of predator–prey interactions are particularly valuable for validating assumptions otherwise inferred from indirect approaches. Our study indicates that tiger sharks may have a limited effect on annual fledgling mortality, suggesting that ecological models may need to be updated with these empirical tiger shark behavior data, which would result in more accurate predictions of ecological processes.

In addition, this study demonstrates the value of maintaining relatively pristine ecosystems to understand ecological processes. Few locations globally have documented tiger shark aggregations in association with seasonal prey pulses (Fitzpatrick et al. 2012; Meyer et al. 2010), highlighting the rare natural phenomenon of considerable ecological significance of the predator–prey dynamics directly observed at Kure Atoll. Continued protection of the Papahānaumokuākea Marine National Sanctuary, including its islands and irreplaceable living laboratories within which to understand ecosystem function and trophic ecology, therefore preserves not only species of conservation concern, but also the opportunity to study long‐standing ecological interactions that may have persisted across evolutionary and ecological timescales.

In conclusion, this study highlights the significance of prey vulnerability and timing of prey availability for shaping predator–prey interactions in predictable resource pulses. Contrary to the traditional notion that prey density defines predator presence and predation risks, our results show that predators may instead respond to periods when prey are most susceptible to capture. Tiger shark sightings within the lagoon preceded peak fledgling densities, indicating that maximum predator occurrence in the survey area occurred before maximum prey availability. Fledglings were subject to the highest predation rate at the beginning of fledging season when inexperienced fledglings were likely most vulnerable. In fact, tiger shark sightings peaked and decreased before fledgling abundance peaked. Our findings illustrate that temporal variation in prey vulnerability and accessibility may be more important than prey quantity in shaping predator behavior.

More broadly, this study represents one of the few available works exploring predation dynamics in predictable, temporary resource pulses by large marine predators. Few places worldwide report on the aggregation of tiger sharks associated with seasonal prey pulses (Fitzpatrick et al. 2012; Meyer et al. 2010), and none have directly quantified the temporal dynamics linking predator occurrence, prey vulnerability, and predation success. By documenting these behavioral interactions across an entire fledging season, our findings demonstrate how behavioral responses to temporally varying prey conditions can generate ecological patterns of predation risk. Our study suggests that temporal variation in prey vulnerability represents an important mechanism linking predator behavior and ecological patterns within pulsed‐resource systems.

## Author Contributions

**Caitlin Dudzik:** conceptualization (equal), data curation (lead), formal analysis (lead), writing – original draft (lead). **Andrew Chin:** conceptualization (equal), supervision (supporting), writing – original draft (supporting). **Eva C. McClure:** formal analysis (supporting), supervision (lead), writing – original draft (supporting).

## Funding

The authors have nothing to report.

## Conflicts of Interest

The authors declare no conflicts of interest.

## Data Availability

All data supporting the findings of this study are published in Zenodo and are publicly available at https://doi.org/10.5281/zenodo.20713907.
